# Effects of Androgen Excess-Related Metabolic Disturbances on Granulosa Cell Function and Follicular Development

**DOI:** 10.3389/fendo.2022.815968

**Published:** 2022-02-14

**Authors:** Baoying Liao, Xinyu Qi, Chuyu Yun, Jie Qiao, Yanli Pang

**Affiliations:** ^1^ Center for Reproductive Medicine, Department of Obstetrics and Gynecology, Peking University Third Hospital, Beijing, China; ^2^ National Clinical Research Center for Obstetrics and Gynecology, Peking University Third Hospital, Beijing, China; ^3^ Key Laboratory of Assisted Reproduction, Peking University, Ministry of Education, Beijing, China; ^4^ Beijing Key Laboratory of Reproductive Endocrinology and Assisted Reproductive Technology, Peking University Third Hospital, Beijing, China; ^5^ Department of Physiology and Pathophysiology, School of Basic Medical Sciences, Peking University, Beijing, China; ^6^ Key Laboratory of Molecular Cardiovascular Science, Peking University, Ministry of Education, Beijing, China

**Keywords:** polycystic ovary syndrome, ovarian dysfunction, metabolic disorders, *in vitro* follicle culture, follicular development

## Abstract

Polycystic ovary syndrome (PCOS) is a common reproductive endocrine disease in women of reproductive age. Ovarian dysfunction including abnormal steroid hormone synthesis and follicular arrest play a vital role in PCOS pathogenesis. Hyperandrogenemia is one of the important characteristics of PCOS. However, the mechanism of regulation and interaction between hyperandrogenism and ovulation abnormalities are not clear. To investigate androgen-related metabolic state in granulosa cells of PCOS patients, we identified the transcriptome characteristics of PCOS granulosa cells by RNA-seq. Gene ontology (GO) and Kyoto Encyclopedia of Genes and Genomes (KEGG) analysis of differentially expressed genes (DEGs) revealed that genes enriched in lipid metabolism pathway, fatty acid biosynthetic process and ovarian steroidogenesis pathway were abnormally expressed in PCOS granulosa cells in comparison with that in control. There are close interactions among these three pathways as identified by analysis of the protein-protein interaction (PPI) network of DEGs. Furthermore, *in vitro* mouse follicle culture system was established to explore the effect of high androgen and its related metabolic dysfunction on follicular growth and ovulation. RT-qPCR results showed that follicles cultured with dehydroepiandrosterone (DHEA) exhibited decreased expression levels of cumulus expansion-related genes (*Has2*, *Ptx3*, *Tnfaip6* and *Adamts1*) and oocyte maturation-related genes (*Gdf9* and *Bmp15*), which may be caused by impaired steroid hormone synthesis and lipid metabolism, thus inhibited follicular development and ovulation. Furthermore, the inhibition effect of DHEA on follicle development and ovulation was ameliorated by flutamide, an androgen receptor (AR) antagonist, suggesting the involvement of AR signaling. In summary, our study offers new insights into understanding the role of androgen excess induced granulosa cell metabolic disorder in ovarian dysfunction of PCOS patients.

## Introduction

PCOS is a common reproductive endocrine disorder in women of reproductive age. Characterized by menstrual disorder, hirsutism and acne induced by hyperandrogenism and polycystic ovarian morphology, PCOS affects 5–10% of women worldwide and is the primary cause of anovulatory infertility in women of reproductive age (1). The ovulatory disorder in women with PCOS is due to follicular development arrest, which is associated with the disorder of hypothalamic gonadotropin secretion and hyperandrogenic environment in ovaries (2). However, the precise network regulation mechanism of follicular development arrest by hyperandrogenism remains unclear.

As an important cell type in follicular formation and ovulation, the function of granulosa cells is closely related to anovulation and metabolic disorder in PCOS (3–6). Mounting of evidence supported that the disruption of follicle development and ovulation process in PCOS is associated with granulosa cell dysfunction. It is reported that the activation of endoplasmic reticulum (ER) stress in granulosa cells participated in promoting follicular atresia and anovulation in DHEA-induced PCOS like mouse model (7). Jin et al. further explored the effect of androgen exposure on the function of granulosa cells and found that testosterone significantly induced ER stress and apoptosis of ovarian granulosa cells *in vitro*, indicating the negative effect of androgen-induced ER stress on follicle development (8). In addition, Li et al. revealed a positive correlation between serum testosterone levels and the expression of autophagy-related genes, suggesting that androgen excess contributed to the activation of autophagy and apoptosis in granulosa cells, which subsequently impairs ovarian function (9, 10). Combined, these results suggested androgens promote apoptosis of granulosa cells in PCOS, while the specific role and mechanism of androgens in regulating follicular growth and ovulation remains unclear.

Metabolic disorders also play a vital role in PCOS pathogenesis. Compared to women with regular ovulation, anovulatory PCOS patients showed obvious dyslipidemia, namely, increased serum cholesterol, triglycerides, and also low-density lipoprotein (LDL) levels and decreased high-density lipoprotein (HDL) levels (11), which were closely related to testosterone levels. In addition, lipid metabolism dysfunction in granulosa cells was also found to contribute to PCOS development (12). In human granulosa cells, oxidized low-density lipoprotein induced cell death and subsequently contributed to ovulatory dysfunction, indicating that disorders of lipid metabolism in granulosa cells may be implicated in PCOS development (13). On the other hand, fatty acid levels were proposed as the predictors of *in vitro* fertilization outcomes for their bi-directional effect on granulosa cell function and oocyte competence. However, fatty acid profiles in PCOS serum and follicular fluid were both found to significantly differ from that in healthy women, suggesting the important role of granulosa cell metabolic disorders in PCOS development (14). Intriguingly, androgen excess has long been demonstrated to participate in the development of various metabolic disorders in PCOS (15). However, the mechanism of how high androgen exposure promotes metabolic disorders in PCOS granulosa cells remains elusive.

As the basic functional unit of the ovary, ovarian follicle consists of oocyte and surrounding granulosa cells. Folliculogenesis is a vital process for the production of fertilizable and developmentally-competent oocyte, which requires the regulation of various signals including, but not restricted to, hormonal regulation, paracrine signals and the bidirectional communication between oocyte and granulosa cells (4, 16, 17). To better understand the biology and regulatory mechanism of follicle development, various culture methods have been established (18–21). *In vitro* follicle culture system which uses alginate as the scaffold to preserve the structural integrity and function of ovarian follicle has been established to explore the dynamic of follicle development (22–24). Furthermore, mature oocyte and live birth were obtained from mouse follicles cultured *in vitro* (25), suggesting the superiority of this system in mimicking folliculogenesis process *in vivo*. In general, *in vitro* follicle culture system enables us to investigate the biology of folliculogenesis and oocyte maturation *in vitro*, which has great significance for fertility preservation and provides an excellent *in vitro* model to investigate the pathophysiology of ovulatory disorder in anovulatory diseases.

Here we performed RNA-seq analysis to comprehensively reveal the hyperandrogenism-induced functional disorders of PCOS granulosa cells. *In vitro* mouse follicle culture system was established to examine the effect of hyperandrogenic exposure on the function of granulosa cells and ovarian follicles, with AR antagonist supplied to figure out the action mode of androgen. Overall, this study provides insights into hyperandrogenism induced abnormal follicle development in PCOS.

## Methods and Materials

### Human Subjects

The study protocol was approved by the Ethics Committee of Peking University Third Hospital according to the Council for International Organizations of Medical Sciences. According to the 2003 Rotterdam criteria, women with PCOS were diagnosed when at least two of the following clinical manifestations occurred: (1) oligo-ovulation and/or anovulation; (2) clinical and/or biochemical hyperandrogenism; and (3) polycystic ovaries. Cushing syndrome, thyroid disease, 21-hydroxylase deficiency, androgen-secreting tumors, congenital adrenal hyperplasia, and hyperprolactinemia should be excluded before the diagnosis of PCOS. Infertile individuals with only tubal occlusion or male azoospermia were recruited as the control subjects. Informed consent has been signed by all participants. The clinical characteristics of enrolled individuals are listed in **Table 1**.

**Table 1:** Clinical characteristics in women with and without PCOS.

	Control (n = 6)	PCOS (n = 6)	*P*-value
Age (year)	30.3 ± 4.63	29.0 ± 4.94	0.6400
Body Mass Index	23.9 ± 1.72	22.7 ± 1.86	0.2658
FSH (mIU/ml)	6.35 ± 1.37	6.35 ± 1.21	0.9896
LH (mIU/ml)	3.09 ± 1.19	11.5 ± 5.77	0.0151
LH/FSH	0.48 ± 0.13	1.94 ± 1.36	0.0467
Estradiol (pmol/ml)	224.58 ± 146.89	219.5 ± 106.6	0.9468
Testosterone (nmol/L)	0.69 ± 0.03	1.90 ± 0.53	0.0026
Androstenedione (nmol/L)	5.37 ± 1.94	16.4 ± 2.71	1.95E-5

FSH, follicle-stimulating hormone; LH, luteinizing hormone. All data are expressed as the mean ± S.E.M. Data were analyzed by two-tailed Student’s t-test.

### Human Granulosa Cell Collection and Culture

We enrolled 6 control and 6 women with PCOS for granulosa cell collection and RNA extraction. Both individuals in control group and women with PCOS were on the first *in vitro* fertilization cycle. Both individuals in control group and women with PCOS were on the first *in vitro* fertilization cycle. A gonadotrophin releasing hormone antagonist protocol was applied for all donors. After 36 h of human chorionic gonadotropin (hCG) administration, follicular fluid was obtained from controls and PCOS women through transvaginal ultrasound-guide follicle aspiration. As described previously (26), human granulosa cells were separated with density gradient centrifugation and culture in DMEM-F12 supplemented with 10% fetal bovine serum (FBS), and 1% penicillin–streptomycin (5,000 U/ml) for 12 h, and subsequently collected for RNA extraction.

### RNA Extraction and RNA Sequencing (RNA-seq) Analysis

Total RNA was extracted from collected human granulosa cells with TRIzol reagent (15596018; Life Technologies) according to the manufacturer’s protocol. Total RNA from two individuals were mixed for RNA-seq. After RNA quantification and qualification, a total amount of 1μg RNA per sample was used as input material for the RNA ample preparation. Sequencing libraries were generated using NEBNext^®^ UltraTM RNA Library Prep Kit for Illumina^®^ (NEB, USA) following manufacturer’s recommendations and index codes were added to attribute sequences to each sample. The clustering of the index-coded samples was performed on a cBot Cluster Generation System using TruSeq PE Cluster Kit v3-cBot-HS (Illumia) according to the manufacturer’s instructions. After cluster generation, the library preparations were sequenced on an Illumina Novaseq platform and 150 bp paired-end reads were generated. Before data analysis, raw data (raw reads) of fastq format were firstly processed through in-house perl scripts for quality control. In this step, clean data (clean reads) were obtained by removing reads containing adapter, reads containing ploy-N and low-quality reads from raw data. At the same time, Q20, Q30, and GC content the clean data were calculated. All the downstream analyses were based on the clean data with high quality. As to data analysis, raw data (raw reads) of fastq format were firstly processed through in-house perl scripts, at the same time, Q20, Q30, and GC content the clean data were calculated. All the downstream analyses were based on the clean data with high quality. Reference genome and gene model annotation files were downloaded from genome website directly. Index of the reference genome was built using Hisat2 v2.0.5 and paired-end clean reads were aligned to the reference genome using Hisat2 v2.0.5. Significant differentially expressed genes were defined by the criteria of FDR q <0.05 and log2(FC) ≥1. Bioinformatic analysis was performed using the OmicStudio tools at https://www.omicstudio.cn/tool. The data presented in the study are deposited in the GEO database, accession number GSE193123.

### *In Vitro* Culture of Mouse Ovarian Follicles

Used for the mechanical separation of secondary follicles were 18 to 21-day-old C57BL/6J mice. Healthy follicles (intact and round oocyte in the central of follicles, with 2–3 layers of granulosa cells surrounded and covered with intact theca cell layer) were selected for culturing. While atretic follicles (follicles with darken granulosa cells) and damaged follicles (follicles with the extrusion of oocyte or granulosa cells) were excluded at the beginning of *in vitro* culture. Healthy follicles were incubated in maintenance media (αMEM [32571036, Sigma-Aldrich] with 1% FBS) for 1 h before encapsulation. As described previously (25, 27), each follicle was capsulated with 0.5% alginate (Sigma-Aldrich) to maintain its architecture, and then cultured with 100 μl growth media (αMEM, 3 mg/ml BSA [B2064, Sigma-Aldrich], 1 mg/ml bovine fetuin [F2379, Sigma-Aldrich], 10 mIU/ml recombinant follicular stimulating hormone, 5 μg/ml insulin, 5 μg/ml transferrin, and 5 μg/ml selenium [I3146, Sigma-Aldrich]) in 96-well plate. DHEA (10 μM; HY-14650; Med Chem Express) was supplied into growth media to mimic hyperandrogenic environment in PCOS ovaries, flutamide (10 μM; F9397; Sigma-Aldrich) was supplied into growth media to block androgen receptor. Half of the growth media was changed every 2 days and the supernatants were collected for estradiol detection after culture. Follicles were imaged by fluorescence microscope at each media change. The average length of two perpendicular measurements from basal lamina to basal lamina in ImageJ was considered as the follicle diameter.

### *In Vitro* Maturation of Mouse Ovarian Follicles

On the 6th day of culturing, follicles were released from alginate beads using alginate lyase (A1603, Sigma-Aldrich) and incubated in maturation media (αMEM with 10% FBS, 1%PS, 1.5 IU/ml human chorionic gonadotropin, 10 ng/ml epidermal growth factor [EGF] [PHG0311, Gibco]) for 18 h. After 18 h of incubation, ruptured follicles, ovulated COCs and oocytes were imaged, and the ovulation rate was calculated by observing the ovulation of 7 follicles. The follicular wall of ovulated follicles was ruptured, with ovulated cumulus–oocyte-complex (COC) around. Oocytes with first polar body extrusion were classified as mature oocytes. Every 3 follicles were collected for RNA extraction using RNeasy Mini Kit (74104, QIAGEN).

### cDNA Synthesis and Quantitative Real-Time PCR Analysis

cDNA was synthesized from 1,000 ng RNA using the RevertAid First cDNA Synthesis Kit (K1622; Thermo Scientific) according to the manufacturer’s protocols. The primers used for Real-time qPCR are listed in **Table 2**. Real-time qPCR was performed in an ABI 7500 real-time PCR system (Applied Biosystems) using SYBR Green PCR Master Mix (Invitrogen). The relative expression level of genes was normalized to those of 18S rRNA in mouse and *ACTIN* in human.

**Table 2:** Primer Sequence used in quantitative real-time PCR analysis.

Target genes	Primer sequence
18S (mouse)	Forward 5’-GAAACGGCTACCACATCCAAGG-3’
	Reverse 5’-GCCCTCCAATGGATCCTCGTTA-3’
*Cyp17a1* (mouse)	Forward 5’-GCCCAAGTCAAAGACACCTAAT-3’
	Reverse 5’-GTACCCAGGCGAAGAGAATAGA-3’
*Cyp19a1* (mouse)	Forward 5’-ATGTTCTTGGAAATGCTGAACCC-3’
	Reverse 5’-AGGACCTGGTATTGAAGACGAG-3’
*Amh* (mouse)	Forward 5’-CCACACCTCTCTCCACTGGTA-3’
	Reverse 5’-GGCACAAAGGTTCAGGGGG-3’
*Gdf9* (mouse)	Forward 5’-TCTTAGTAGCCTTAGCTCTCAGG-3’
	Reverse 5’-TGTCAGTCCCATCTACAGGCA-3’
*Bmp15* (mouse)	Forward 5’-TCCTTGCTGACGACCCTACAT-3’
	Reverse 5’-TACCTCAGGGGATAGCCTTGG-3’
*Has2* (mouse)	Forward 5’-TGTGAGAGGTTTCTATGTGTCCT-3’
	Reverse 5’-ACCGTACAGTCCAAATGAGAAGT-3’
*Ptx3* (mouse)	Forward 5’-CCTGCGATCCTGCTTTGTG-3’
	Reverse 5’-GGTGGGATGAAGTCCATTGTC-3’
*Adamts1* (mouse)	Forward 5’-CATAACAATGCTGCTATGTGCG-3’
	Reverse 5’-TGTCCGGCTGCAACTTCAG-3’
*Tnfaip6* (mouse)	Forward 5’-GGGATTCAAGAACGGGATCTTT-3’
	Reverse 5’-TCAAATTCACATACGGCCTTGG-3’
*Hmgcr* (mouse)	Forward 5’-AGCTTGCCCGAATTGTATGTG-3’
	Reverse 5’-TCTGTTGTGAACCATGTGACTTC-3’
*Fads2* (mouse)	Forward 5’-GATGGCTGCAACATGACTATGG-3’
	Reverse 5’-GCTGAGGCACCCTTTAAGTGG-3’
*Fasn* (mouse)	Forward 5’-GGAGGTGGTGATAGCCGGTAT-3’
	Reverse 5’-TGGGTAATCCATAGAGCCCAG-3’
*Srebf1* (mouse)	Forward 5’-GCAGCCACCATCTAGCCTG-3’
	Reverse 5’-CAGCAGTGAGTCTGCCTTGAT-3’
*Insig1* (mouse)	Forward 5’-CACGACCACGTCTGGAACTAT-3’
	Reverse 5’-TGAGAAGAGCACTAGGCTCCG-3’
*Ldlr* (mouse)	Forward 5’-AGTGGCCCCGAATCATTGAC-3’
	Reverse 5’-CTAACTAAACACCAGACAGAGGC-3’
*Acss2* (mouse)	Forward 5’-AAACACGCTCAGGGAAAATCA-3’
	Reverse 5’-ACCGTAGATGTATCCCCCAGG-3’
*Lss* (mouse)	Forward 5’-TCGTGGGGGACCCTATAAAAC-3’
	Reverse 5’-CGTCCTCCGCTTGATAATAAGTC-3’
*ACTB* (human)	forward 5’-GAGCACAGAGCCTCGCCTTT-3’
	reverse 5’-TCATCATCCATGGTGAGCTGG-3’
*HMGCR* (human)	Forward 5’-TGATTGACCTTTCCAGAGCAAG-3’
	Reverse 5’-CTAAAATTGCCATTCCACGAGC-3’
*FASN* (human)	Forward 5’-TGATTGACCTTTCCAGAGCAAG-3’
	Reverse 5’-CTAAAATTGCCATTCCACGAGC-3’
*SREBF1* (human)	Forward 5’-CGGAACCATCTTGGCAACAGT-3’
	Reverse 5’-CGCTTCTCAATGGCGTTGT-3’
*SCD* (human)	Forward 5’-TTCCTACCTGCAAGTTCTACACC-3’
	Reverse 5’-CCGAGCTTTGTAAGAGCGGT-3’
*INSIG1* (human)	Forward 5’-GCCTACTGTACCCCTGTATCG-3’
	Reverse 5’-TGGTTAATGCCAACAAAAACTGC-3’
*LDLR* (human)	Forward 5’-ACGGCGTCTCTTCCTATGACA-3’
	Reverse 5’-CCCTTGGTATCCGCAACAGA-3’
*LSS* (human)	Forward 5’-GTACGAGCCCGGAACATTCTT-3’
	Reverse 5’-CGGCGTAGCAGTAGCTCAT-3’
*SCD5* (human)	Forward 5’-TGCGACGCCAAGGAAGAAAT-3’
	Reverse 5’-CCTCCAGACGATGTTCTGCC-3’
*FADS2* (human)	Forward 5’-GACCACGGCAAGAACTCAAAG-3’
	Reverse 5’-GAGGGTAGGAATCCAGCCATT-3’
*ACSS2* (human)	Forward 5’-AAAGGAGCAACTACCAACATCTG-3’
	Reverse 5’-GCTGAACTGACACACTTGGAC-3’

### Screening of Hub Genes

The PPI network of the STRING database was applied to reveal the relationship between the DEGs. Then, the network relationship file was downloaded, and the top 10 hub genes were identified in accordance with Cytoscape 3.6.1 and its plug-in (degrees ranking of cytoHubba).

### Statistical Analysis

The data are shown as Mean ± SEM. For parametric data, statistical analyses were carried out using SPSS version 23 by two-tailed Student’s t-test or one-way ANOVA with Tukey’s *post hoc* test and represented with GraphPad Prism version 8.0 (GraphPad Software). For nonparametric data, statistical analyses were carried out by the two-tailed Mann–Whitney U-test or the Kruskal–Wallis test followed by Dunn’s *post hoc* test. *P <0.05, ** P <0.01, *** P <0.001; ^#^P <0.05, ^##^ P <0.01, ^###^ P <0.001.

## Results

### Identification of the Transcriptional Landscapes of PCOS Granulosa Cells

**Figure 1:**
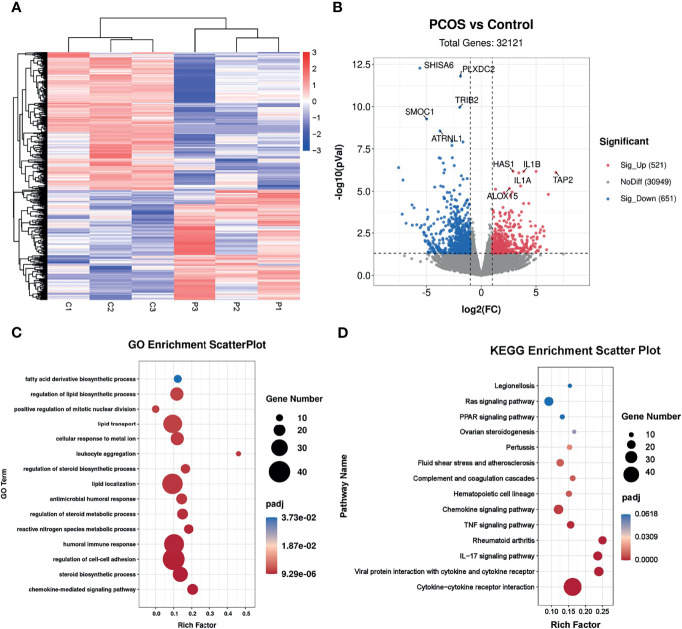
Identification of the transcriptional landscapes of PCOS granulosa cells. **(A)** Heatmap of differential expressed genes in the control and PCOS granulosa cells. **(B)** Volcano plot showing transcriptomic landscapes in control and PCOS group. Significant differentially expressed genes (DEGs) were examined with padj (adjusted p-value) < 0.05. Meanwhile, log2 fold change >1 was set as the threshold for significant differential expression. **(C)** GO enrichment analysis showing 15 pathways from the top 50 pathways enriched in PCOS granulosa cells; **(D)** KEGG pathway analysis showing the top 15 pathways involved in PCOS pathogenesis.

To investigate the potential effects of hyperandrogenism on granulosa cells from PCOS patients, we performed RNA-seq analysis on granulosa cells using a PCOS cohort with significantly upregulated serum testosterone and androstenedione levels in comparison with BMI-matched individuals in control group. The raw data of RNA-seq analysis were firstly processed for quality control and downstream analyses were based on the clean data with high quality. According to the heatmap, the transcripts of PCOS granulosa cells evidently differed from that of control (**Figure 1A**). In comparison with control, a total of 1,172 genes were significantly changed in PCOS granulosa cells, of which 521 genes were upregulated and 651 genes were downregulated respectively in PCOS granulosa cells. Genes encoding inflammatory factors including interleukin-1beta (*IL1B*) and interleukin-1alpha (*IL1A*) showed remarkable upregulation in PCOS granulosa cells; besides, the expression of arachidonate 15-lipoxygenase (*ALOX15*) was also evidently upregulated. ALOX15 is a member of the lipoxygenase family, which plays an important role in polyunsaturated fatty acid metabolism, indicating that metabolic disorders may have occurred in PCOS granulosa cells (**Figure 1B**). To investigate the specific signaling pathway involved in PCOS pathogenesis, we performed GO enrichment analysis and KEGG analysis of all DEGs and found that DEGs were evidently enriched in immune related pathways, namely, chemokine-mediated signaling pathway and humoral immune response, indicating the possible role of immune factors in PCOS development. In addition, fatty acid biosynthetic process and lipid metabolism-related pathways were also enriched in PCOS granulosa cells (**Figure 1C**). Furthermore, more than one fifth of the top 50 pathways enriched through GO analysis were metabolic related pathways (**Table 3**), suggesting that the impairment of metabolic process, especially fatty acid biosynthetic and lipid metabolism pathways may play a vital role in promoting granulosa cells dysfunction in PCOS. Similarly, KEGG analysis of DEGs also suggested the misexpression of genes related to chemokine signaling pathway in PCOS granulosa cells. Moreover, PPAR signaling pathway were enriched in KEGG analysis, suggesting that metabolic state in PCOS granulosa cells evidently differed from that in control, and the enrichment of DEGs in ovarian steroidogenesis may further support the abnormal secretion of steroid hormones by granulosa cells in women with PCOS (**Figure 1D**). Overall, these results indicated that lipid metabolism disorders are important characteristics of granulosa cells in PCOS patients.

**Table 3:** GO enrichment analysis of differential expressed genes (metabolic-related pathways in the top 50 GO terms).

GOID	Description	*p-*value	padj	Gene Name
GO:0006694	steroid biosynthetic process	1.14E−07	3.30E−05	*IL1B/SCAP/NR1H4/SREBF1/HMGCR/FAXDC2/PRLR/TM7SF2/APOE/HSD11B2/ACOX2/CYP19A1/INSIG1/FASN/CYP11B1/LSS/BMP6/CYP1A1/DHCR24/SCD/CYP11A1/BMP2/FDXR/CYP27B1/SCARB1/WNT4/ABCB11*
GO:0019218	regulation of steroid metabolic process	4.22E−06	0.000985	*IL1B/SCAP/NR1H4/APOC1/SREBF1/HMGCR/TM7SF2/APOE/EPHX2/INSIG1/FASN/LSS/BMP6/LDLR/SCD/BMP2/CYP27B1/WNT4*
GO:0010876	lipid localization	7.08E−06	0.001505	*ABCA5/IL1B/NR1H4/APOC1/ZC3H12A/IL6/LCN12/ABCA10/CLU/KCNN4/C3/SERPINA5/ATP8A2/ABCA9/TNFAIP8L3/ATP8B3/APOE/EDN1/PRKN/SLC51A/MTTP/SPNS3/GULP1/CYP19A1/SLC27A6/SLCO1A2/OSBPL10/PLA2G12A/FABP6/BMP6/SPNS2/LDLR/PNLIP/SCARB1/ABCA13/ABCB11/ANO9/ACSL4*
GO:0050810	regulation of steroid biosynthetic process	7.37E−06	0.001505	*IL1B/SCAP/NR1H4/SREBF1/HMGCR/TM7SF2/APOE/INSIG1/FASN/LSS/BMP6/SCD/BMP2/CYP27B1/WNT4*
GO:0006869	lipid transport	1.23E−05	0.002105	*ABCA5/IL1B/NR1H4/APOC1/LCN12/ABCA10/CLU/KCNN4/SERPINA5/ATP8A2/ABCA9/TNFAIP8L3/ATP8B3/APOE/EDN1/PRKN/SLC51A/MTTP/SPNS3/GULP1/CYP19A1/SLC27A6/SLCO1A2/OSBPL10/PLA2G12A/FABP6/BMP6/SPNS2/LDLR/PNLIP/SCARB1/ABCA13/ABCB11/ANO9/ACSL4*
GO:0046890	regulation of lipid biosynthetic process	1.29E−05	0.002105	*IL1B/SCAP/NR1H4/APOC1/SREBF1/HMGCR/SPHK1/C3/TM7SF2/APOE/PTGS2/INSIG1/FASN/SMPD3/LSS/BMP6/LDLR/SCD/BMP2/CYP27B1/SCARB1/WNT4/PDGFB*
GO:0008202	steroid metabolic process	1.47E−05	0.002328	*IL1B/SCAP/NR1H4/APOC1/SREBF1/HMGCR/FAXDC2/DHRS9/PRLR/TM7SF2/APOE/HSD11B2/EPHX2/ACOX2/CYP19A1/INSIG1/FASN/CYP11B1/LSS/BMP6/CYP1A1/DHCR24/LDLR/SCD/CYP11A1/BMP2/WWOX/FDXR/CYP27B1/SCARB1/WNT4/ABCB11*
GO:0016125	sterol metabolic process	2.91E−05	0.003445	*SCAP/NR1H4/APOC1/SREBF1/HMGCR/FAXDC2/TM7SF2/APOE/EPHX2/CYP19A1/INSIG1/FASN/CYP11B1/LSS/DHCR24/LDLR/SCD/CYP11A1/FDXR/SCARB1*
GO:0090181	regulation of cholesterol metabolic process	3.72E−05	0.003965	*SCAP/NR1H4/SREBF1/HMGCR/TM7SF2/APOE/EPHX2/FASN/LSS/LDLR/SCD*
GO:1902652	secondary alcohol metabolic process	3.91E−05	0.004082	*SCAP/NR1H4/APOC1/SREBF1/HMGCR/TM7SF2/APOE/EPHX2/INSIG1/FASN/CYP11B1/LSS/DHCR24/LDLR/SCD/CYP11A1/FDXR/CYP27B1/SCARB1*
GO:0019216	regulation of lipid metabolic process	4.80E−05	0.004903	*IL1B/SCAP/NR1H4/APOC1/SREBF1/HMGCR/LGALS12/SPHK1/C3/TM7SF2/TNFAIP8L3/APOE/PSAPL1/PTGS2/EPHX2/CCR7/INSIG1/CCKBR/FASN/NPAS2/PLPP1/SMPD3/LSS/BMP6/CYP1A1/SOCS1/LDLR/SCD/MTMR2/BMP2/CYP27B1/ANKRD1/SCARB1/WNT4/EPHA8/PDGFB*

### Interaction Between Metabolic Disorders and Ovarian Steroidogenesis in PCOS Granulosa Cells

**Figure 2:**
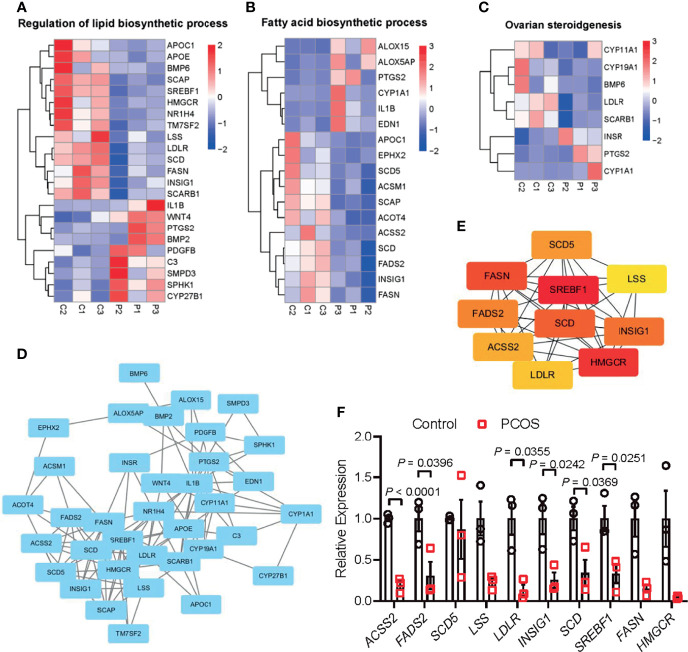
Interaction between metabolic disorders and ovarian steroidogenesis in PCOS granulosa cells. **(A)** Heatmap of differential expressed genes involved in the fatty acid biosynthetic process, **(B)** lipid metabolism, **(C)** ovarian steroidogenesis in the PCOS group and control group. **(D)** Protein–protein interaction (PPI) network between fatty acid biosynthetic process, lipid metabolism, and ovarian steroidogenesis. **(E)** Ten hub genes (*SREBF1*, *HMGCR*, *FASN*, *SCD*, *INSIG1*, *FADS2*, *SCD5*, *ACSS2*, *LDLR*, and *LSS*) in the PPI network. **(F)** mRNA expression levels of 10 hub genes (*SREBF1*, *HMGCR*, *FASN*, *SCD*, *INSIG1*, *FADS2*, *SCD5*, *ACSS2*, *LDLR*, and LSS) in granulosa cells from control and women with PCOS, N = 3. Data were analyzed by two-tailed Student’s t-test **(F)**. All data are presented as the Mean ± SEM.

Given the significant enrichment of DEGs in metabolic-related pathways in PCOS granulosa cells, we further analyzed the specific genes involved in these pathways. Among genes involved in the regulation of lipid biosynthetic process, *APOC1*, *APOE*, *SCAP*, *SREBF1*, and *LDLR*, which played a vital role in lipid transportation and metabolism, were significantly downregulated in PCOS patients (**Figure 2A**); some of these genes were also enriched in fatty acid biosynthetic process. *ALOX15* and *ALOX5AP*, members of lipoxygenases which promote oxygenation of poly-unsaturated fatty acids and produce pro-inflammatory agents, were upregulated in PCOS granulosa cells. Misexpression of *CYP1A1*, which encodes a member of the cytochrome P450 superfamily of enzymes, also contributed to the difference in fatty acid biosynthesis process in healthy control and women with PCOS (**Figure 2B**). DEGs involving *CYP11A1* and *CYP19A1* were significantly enriched in ovarian steroidogenesis pathway, and this may be related to abnormal steroid hormone synthesis in PCOS (**Figure 2C**). Furthermore, the PPI network of these pathways identified a close interaction between ovarian steroidogenesis, lipid biosynthetic and fatty acid biosynthetic process in PCOS granulosa cells. This network had 36 nodes and 125 interactions, indicating that abnormal ovarian steroidogenesis was evidently correlated with lipid metabolism and fatty acid biosynthetic process in PCOS granulosa cells (**Figure 2D**). Besides, 10 hub genes, including *SREBF1*, *HMGCR*, *FASN*, *SCD*, *INSIG1*, *FADS2*, *SCD5*, *ACSS2*, *LDLR*, and *LSS*, were identified by Cytohubba (**Figure 2E**), and the RT-qPCR result indicated the decreased expression of these 10 hub genes, among which *SREBF1*, *SCD*, *INSIG1*, *FADS2*, *ACSS2*, and *LDLR* were significantly decreased in granulosa cells from women with PCOS, which was consistent with the RNA-Seq result (**Figure 2F**). These results suggested that the impairment of ovarian steroidogenesis and lipid metabolism interact and may contribute to PCOS development.

### DHEA Impaired Mouse Follicular Growth, Steroidogenesis and Lipid Metabolism *In Vitro*

**Figure 3:**
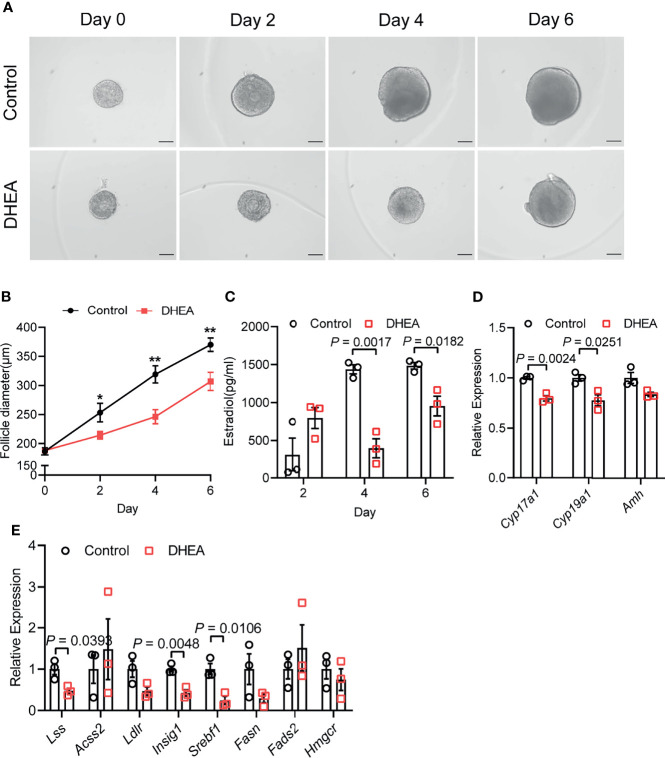
DHEA impaired mouse follicular growth and steroidogenesis *in vitro*. **(A)** Representative micrograph of mouse follicles cultured *in vitro*. **(B)** Follicle diameters in control and DHEA group, N = 9. **(C)** Estradiol levels in the supernatant of control and DHEA-treated follicles, N = 3. **(D)** mRNA expression levels of *Cyp17a1*, *Cyp19a1*, and *Amh* in follicles cultured *in vitro*, N = 3. **(E)** mRNA expression levels of *Lss*, *Acss2*, *Ldlr*, *Insig1*, *Srebf1*, *Fasn*, *Fads2*, and *Hmgcr* in follicles cultured *in vitro*, N = 3. Scale bar: 100 μm. Data were analyzed by two-tailed Student’s t-test **(B–E)**. All data are presented as the Mean ± SEM. *P < 0.05, **P < 0.01.

As mentioned above, granulosa cells from women with PCOS were characterized by the disorder of ovarian steroidogenesis and lipid metabolism, whether the dysfunction of granulosa cells may be related to the impairment of follicle growth and ovulation in PCOS remains unclear. To explore the effect of high androgen exposure-induced lipid metabolism disorder on follicular development, we established an *in vitro* follicle culture system and DHEA was added to simulate the hyperandrogenic environment in PCOS patients. We observed that in the process of *in vitro* culture, separated secondary follicles in control group gradually grew, oocytes moved to one side of the follicle, thus forming antral in the other side of the follicles (**Figure 3A**). After 6 days of *in vitro* culture, follicle diameter could increase from 180 to 360 μm; while supplying DHEA significantly inhibited follicle growth, as the follicles are blocked at 300 μm in diameter (**Figure 3B**). In addition, steroidogenesis process was also impaired by DHEA as estradiol (E2) levels in the supernatant of DHEA-treated follicles was lower than that in control group during 6 days of culturing (**Figure 3C**). E2 is an important steroid hormone synthesized by granulosa cells in growing follicles. It supports the development of ovarian follicles; besides, the elevation of E2 concentrations triggers the LH surge *via* positive feedback effect system, thus inducing ovulation. The decreased E2 levels in DHEA-treated follicles may indicate the impairment of follicle development and ovulation process. We further explored the expression of gene associated with steroid hormone synthesis in follicles. RT-qPCR result showed that *Cyp17a1* and *Cyp19a1*, genes encoding enzymes responsible for the key step in the biosynthesis of androgen and estrogen respectively, were evidently decreased by DHEA (**Figure 3D**), indicating the impairment of steroidogenesis in DHEA-treated follicles. Moreover, mRNA expression levels of hub genes identified from the PPI network of lipid metabolism, fatty acid biosynthesis and ovarian steroidogenesis were investigated in DHEA-treated follicles. The RT-qPCR result indicated the evident decreased expression of *Lss*, *Insig1*and *Srebf1*, which suggested the impairment of lipid metabolism in DHEA-treated follicles and further verified that androgen excess had metabolically harmful effect on granulosa cells (**Figure 3E**). Overall, these results suggested that DHEA treatment induced failure of ovarian steroidogenesis and lipid metabolism, which may subsequently contribute to the disruption of follicle development.

### Supplementation of DHEA Inhibited Ovulation *via* Obstructing Cumulus Expansion

**Figure 4:**
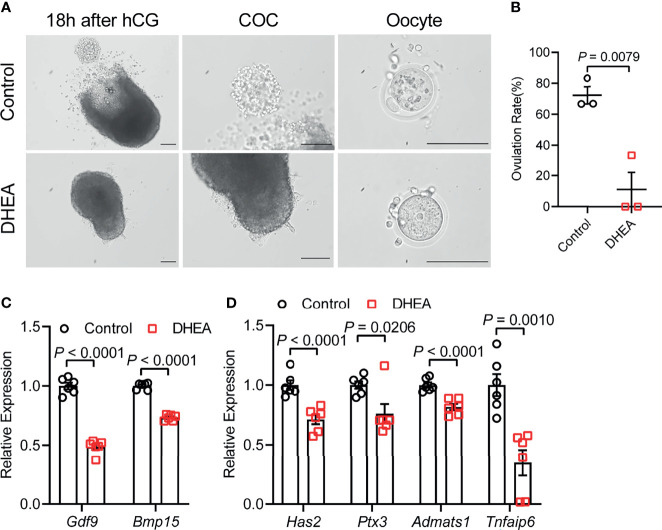
Supplementation of DHEA inhibited ovulation *via* obstructing cumulus expansion. **(A)** The representative micrograph of follicles, ovulated COCs and oocytes after 18 h of maturation. Oocyte with first polar body extrusion was classified as mature oocyte. **(B)** Ovulation rate of *in vitro* cultured follicles, N = 3. **(C)** mRNA expression levels of *Gdf9*, *Bmp15* and **(D)** *Has2*, *Ptx3*, *Tnfaip6* and *Adamts1* in follicles cultured *in vitro*, N = 6. Scale bar: 100 μm. Data were analyzed by two-tailed Mann–Whitney U-test or the Kruskal–Wallis test followed by Dunn’s *post hoc* test **(B)** and two-tailed Student’s t-test **(C, D)**. All data are presented as the Mean ± SEM.

To further investigate the effect of DHEA on ovulation, follicles were released from alginate beads on day 6 and culture with hCG for 18 h. After hCG treatment, we observed that the follicular wall was ruptured, with ovulated cumulus–oocyte-complex (COC) around the ruptured follicle; while no follicular rupture occurred in DHEA treated follicles. We also assessed maturity of follicular oocyte after hCG treatment in two groups. The first polar body extrusion was observed in oocytes denuded from ovulated COCs in control group, whereas oocyte from DHEA group was immature oocyte with germinal vesicle (**Figure 4A**), indicating the inhibition of DHEA on ovulation and oocyte maturation. Statistical result showed that the ovulation rate in DHEA treated follicles was significantly decreased (**Figure 4B**), which was consistent with the result of morphological observation. Ovulation is a complicated process, and the interaction between oocyte and surrounding cumulus cells is important for normal ovulation process. As oocyte secreted factors, GDF9 and BMP15 act on follicular cells adjacent to oocytes to modulate granulosa cells functions (17). GDF9 and BMP15 levels have a positively relationship with oocyte maturation (28). In the present study, *Gdf9* and *Bmp15* mRNA levels were significantly inhibited in DHEA-treated mouse follicles (**Figure 4C**). Additionally, expansion of the COC is essential to ovulation and female fertility (29), the mRNA levels of cumulus expansion related genes, namely, *Has2*, *Ptx3*, *Adamts1*, and *Tnfaip6* were all decreased in follicles treated with DHEA (**Figure 4D**), thus DHEA could inhibit ovulation by suppressing oocyte maturation and cumulus expansion.

### Flutamide Reversed DHEA-Induced Impairment of Follicle Growth and Ovulation Through Blocking AR

**Figure 5:**
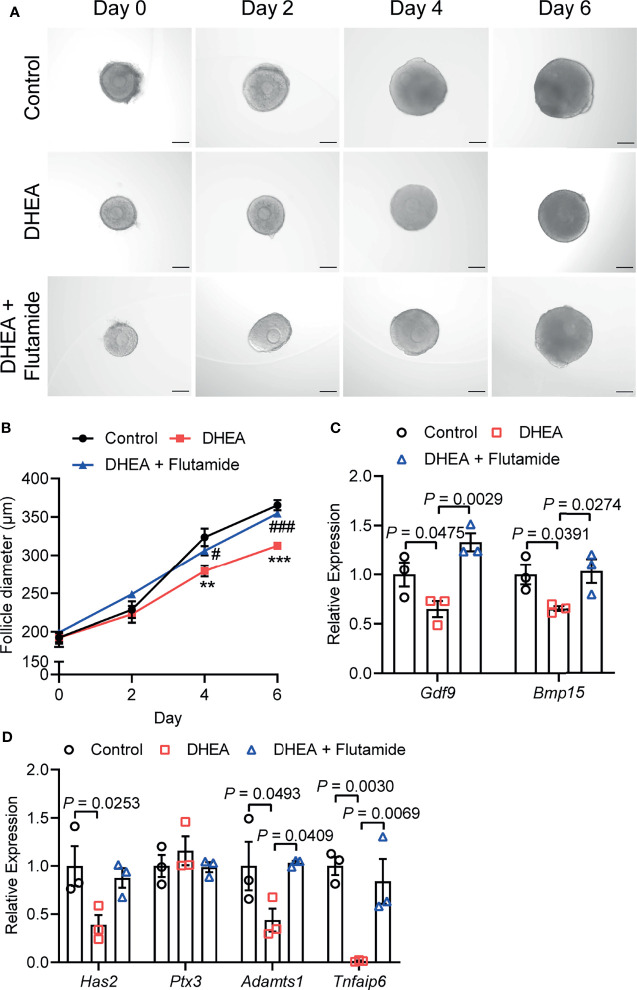
Flutamide reversed DHEA-induced impairment of mouse follicular growth and ovulation *in vitro*. **(A)** Representative micrograph of mouse follicles cultured *in vitro*. **(B)** Follicle diameters in control, DHEA and DHEA + Flutamide group, N = 7. **P < 0.01, ***P < 0.001 versus Control; ^#^P < 0.05, ^###^P < 0.001 versus DHEA. **(C)** mRNA expression levels of *Gdf9*, *Bmp15* and **(D)** *Has2*, *Ptx3*, *Tnfaip6* and *Adamts1* in follicles cultured *in vitro*, N = 3. Scale bar: 100 μm. Data were analyzed by one-way ANOVA with Tukey’s *post hoc* test **(B–D)**. All data are presented as the Mean ± SEM.

AR is considered as key mediators of androgen actions and play an important role in the development of PCOS (30, 31). As a competitive inhibitor of AR, flutamide exhibited therapeutic effect on reproductive and metabolic disorders in PCOS (32). To demonstrate the role of AR signaling in androgen excess induced failure of follicle development and ovulation, flutamide was supplied to DHEA-treated mouse follicles which grew significantly slower than follicles in control group. Flutamide evidently improved follicle growth as the follicle diameter was significantly higher than that in DHEA-treated only group at days 4 and 6 (**Figures 5A, B**). In addition, mRNA expression levels of *Gdf9* and *Bmp15* were significantly decreased in DHEA-treated follicles, while the supply of flutamide significantly reversed the inhibition of DHEA (**Figure 5C**). Furthermore, the blockage of ovulation in follicles cultured with DHEA was also ameliorated by flutamide. The expression of ovulation-related genes *Adamts1* and *Tnfaip6* in follicles was disrupted by DHEA, which was significantly improved by flutamide (**Figure 5D**), indicating the involvement of AR signaling in DHEA induced ovulation disorders. Overall, these results supported that androgen excess induced impairment of follicle growth and ovulation is AR-driven.

## Discussion

PCOS is a complicated reproductive and endocrine syndrome which impairs female fertility. The interaction between hyperandrogenism and metabolic disorders plays an important role in PCOS pathogenesis. In the present study, we mainly focus on the effect of androgen on metabolic dysfunction in PCOS granulosa cells. We analyzed the transcriptome characteristics of PCOS granulosa cells by RNA-seq analysis. The results of GO and KEGG pathway analysis showed that ovarian steroidogenesis, lipid metabolism, and fatty acid biosynthetic pathways were significantly enriched and closely interacted with each other in PCOS granulosa cells. To demonstrate the impact of androgen excess induced lipid metabolism and steroidogenesis disorders in granulosa cells on ovulatory disruption in PCOS, mice ovarian follicles were separated and cultured *in vitro* with DHEA supplementation. It turns out that DHEA supplementation inhibited follicle growth and steroid hormone synthesis *in vitro*; in addition, the ovulation process and oocyte maturation were also impaired by DHEA. Furthermore, the blockage of AR signalling reversed the inhibition of follicle growth and ovulation by DHEA. Combined, we have characterized the transcriptome of PCOS granulosa cells, and further identified the possible ovulation-inhibited effect of altered ovarian steroidogenesis and metabolic disorders in granulosa cells.

**Figure 6:**
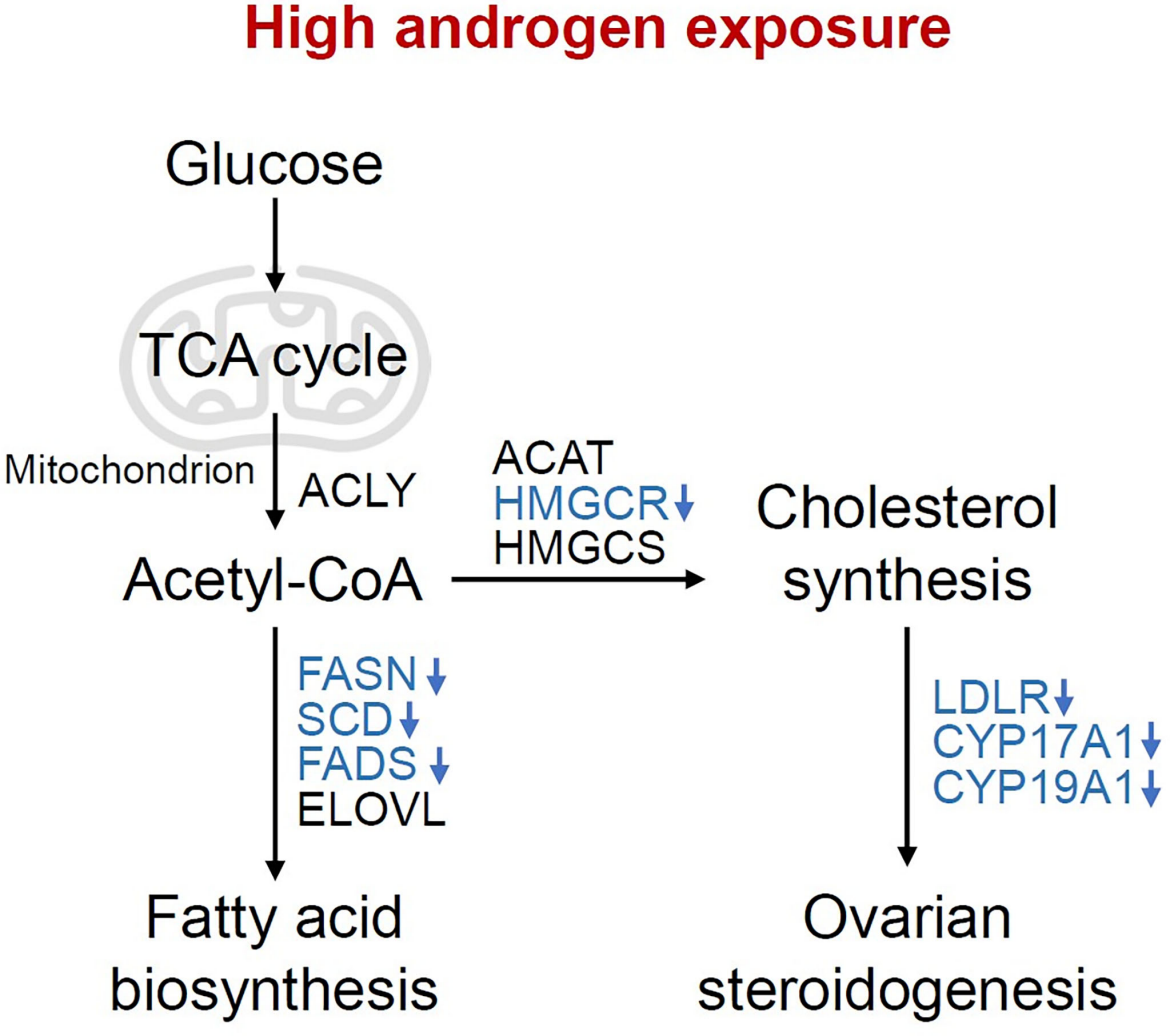
Changes of metabolic pathways in granulosa cells under high androgen exposure. Overview of the pathways and enzymes involved in the synthesis of fatty acids, cholesterol and ovarian steroid hormone. The enzymes enriched in the present study are indicated in blue. TCA cycle, tricarboxylic acid cycle; ACLY, ATP-citrate lyase; ACAT, acetyl-CoA acetyltransferase; HMGCR, 3-hydroxy-3-methylglutaryl-CoA reductase; HMGCS, 3-hydroxy-3-methylglutaryl-CoA synthase; FASN, fatty acid synthase; SCD, stearoyl-CoA desaturase; FADS, fatty acid desaturase; ELOVL, fatty acid elongase; LDLR, Low density lipoprotein receptor; CYP17A1, Cytochrome P450 17A1; CYP19A1, Cytochrome P450 19A1.

Reproduction is closely connected with metabolic status, as oocyte development process requires the supplementation of various nutrients. According to the analysis of the dynamic metabolome profiles in oocytes during *in vivo* maturation, lipid and fatty acid metabolism played a vital role in oocyte meiotic process (5). Additionally, the bi-directional interaction between oocyte and granulosa cells has long been proved to play a key role in oocyte growth and maturation (4, 33), indicating the possible correlation between granulosa cells metabolic status and oocyte maturation, which is less investigated in previous studies. In the present study, we compared the transcriptome of granulosa cells from healthy women and women with PCOS, and found that metabolic process and ovarian steroidogenesis were significantly impaired in PCOS granulosa cells through GO and KEGG pathway analysis. Although metabolic pathways were not ranking high, the proportion of metabolic related pathways in top 50 GO terms were relatively large. Lipid metabolism and fatty acid biosynthetic process were significantly enriched in PCOS granulosa cell. More specific roles that androgen-induced metabolic disorders played in granulosa cells dysfunction were further demonstrated in the PPI network. Among the 10 hub genes we identified through Cytoscape, the expression of 3-hydroxy-3-methylglutaryl-CoA reductase (HMGCR), a rate-limiting enzyme catalyzing cholesterol production, was decreased in human PCOS granulosa cells and prenatally hyperandrogenized animals (34), which may be caused by the feedback inhibition of elevated androgen levels in PCOS (35). Besides, the decline of fatty acid desaturase genes 2 (*FADS2*) expression was also found in women with PCOS, which was correlated with altered androgen levels and dyslipidemia (36). Mediating the uptake of LDL by ovarian follicle cells, *LDLR* was downregulated in PCOS granulosa cells, which is consistent with previous studies (37, 38). In addition, lipid content in granulosa and cumulus cells may affect likelihood of pregnancy, loss of function of *LDLR* resulted in impaired lipid uptake and extracellular lipid accumulation, thus leading to hyperlipidemia and poor fertility in mice (39). Overall, our results showed that abnormal lipid metabolism and fatty acid synthetic pathways were closely related to the development of PCOS, while the specific mechanism underlying how these metabolic disorders contributed to ovulatory disorders in PCOS remains elusive. Actually, most of genes involved in these metabolic disorders in PCOS granulosa cells catalyzed fatty acid biosynthesis, cholesterol synthesis and ovarian steroidogenesis, besides, they were all downregulated under hyperandrogenic environment (**Figure 6**), indicating that the synthesis of fatty acid and cholesterol may be inhibited in PCOS granulosa cells, which may finally contribute to the impairment of oocyte maturation. However, there was no clue indicating changes in the degradation of fatty acid and cholesterol, the true state of fatty acid and cholesterol metabolism in PCOS still needs to be explored.

In terms of the effect of androgen on lipid metabolism, Abruzzese et al. established a prenatally hyperandrogenized rat model and clarified the effect of prenatal androgen exposure on ovarian lipid metabolism (34). Sun et al. compared the steroid and metabolic parameters between women with PCOS and healthy women and found the significantly increased serum androgen levels and upregulated lipid profiles in women with PCOS, which is consistent with previous studies; besides, the cholesterol level was also upregulated in PCOS offspring, indicating that lipid disorders, just like hyperandrogenism and insulin resistance, is a heritable clinical manifestation which may participated in the pathophysiology of PCOS from fetal stage (40). These findings suggested that abnormal lipid metabolism plays an important role in shaping the metabolic and reproductive characteristics in offspring of women with PCOS, in which androgen may participated as well. Recently, Pan et al. reported the reduction of global DNA methylation of PCOS granulosa cells in comparison with granulosa cells from healthy control, which may contributed to the abnormal expression of lipid and steroid synthesis genes for the hypomethylation of their promoters, thus providing a new insight into the possible mechanism that mediating the impact of androgen on lipid and steroid synthesis in granulosa cells (41).

Oocyte and surrounding granulosa cells and theca cells consist of ovarian follicle, the basic functional unit of ovary. *In vitro* follicle culture system provides an opportunity for studying the independent effect of androgen excess on follicle development and ovulation. By adding DHEA into the *in vitro* culture system of mice follicles, we found that lipid metabolism and steroid hormone synthesis in follicles was significantly inhibited by DHEA; additionally, the oocyte maturation-related gene expression was also downregulated after DHEA supplementation, and also cumulus expansion-related genes. Overall, these results indicated that lipid and fatty acid metabolic dysfunction could impair the physiological function of ovarian granulosa cells, which subsequently contribute to the disruption of oocyte maturation and ovulation. During the oocyte maturation process, beta-oxidation of fatty acid provides an important source of ATP for maturing oocyte. Besides, 7 out of 8 enzymes catalyzing fatty acid degradation were upregulated in oocyte meiosis (5), indicating that the increasing utilization of fatty acid plays an important role in promoting oocyte maturation. Furthermore, the follicular microenvironment plays a vital role in modulating oocyte maturation and competence. Fatty acid composition in follicular fluid and granulosa cells could influence oocyte quality (42, 43). In the present study, we found that genes encoding enzymes involved in fatty acid biosynthesis were downregulated in PCOS granulosa cells, which may alter the fatty acid profiles in granulosa cells, thus leading to insufficient energy supply for oocyte maturation. Overall, androgen exposure significantly influenced the metabolic status in granulosa cells from women with PCOS and impaired follicle growth and oocyte maturation, while the underlying molecular mechanism remains to be explored in the future.

AR is widely expressed throughout the body and plays an important role in the pathogenesis of PCOS (31, 44). Extra- and intra-ovarian AR actions both contribute to PCOS development. It is reported that DHT promoted the development of adipocytes hypertrophy and the decrease of adiponectin levels, which was disrupted in AR knockout mice (31). Besides, AR signaling also plays a vital role in mediating the effect of androgen in liver lipid metabolism, as hepatic AR-knockout mice was characterized by hepatic steatosis and insulin resistance (45). In addition, AR is expressed by ovarian theca cells, granulosa cells, and oocyte (46), and granulosa cell specific AR knockout mice exhibited estrous cycle disorder and decreased fertilization rate (47). As a specific androgen antagonist, flutamide competitively inhibits androgen receptors and performs a direct blockage of androgenic effect (48). In the present study, flutamide was found to ameliorate DHEA-induced impairment of follicle development and ovulation, which further supported the direct inhibition of androgen excess on follicle development and suggested the involvement of AR signalling in androgen-induced lipid metabolism and fatty acid synthetic disorders in granulosa cell, which provides new insights into the role of AR-driven metabolic dysfunction of granulosa cells in PCOS pathogenesis.

Some limitations in our study, namely, limited number of clinical samples from both control and women with PCOS, and the functional differences between follicles cultured *in vitro* and follicles grown *in vivo* should be taken into account. Given the heterogeneity of clinical manifestations and the complexity of pathogenesis in PCOS, it is hard to fully mimic the characteristics of PCOS *in vitro*. In the current study, DHEA treatment simulate the hyperandrogenic follicular environment of PCOS ovaries *in vitro*, leading to impaired steroid hormone synthesis, anovulation, and disruption of oocyte maturation, which were partly consistent with the ovarian dysfunctions represented in PCOS patients (49). Taken together, our *in vitro* follicle culture system provided a new possibility for establishing the *in vitro* model of PCOS follicle development, and more evidences are needed to confirm this *in vitro* model.

## Conclusion

In summary, ovarian dysfunction is the main cause for infertility in women with PCOS, and the role of hyperandrogenism in promoting ovarian dysfunction cannot be overlooked. Our study illuminates the transcript characteristics of PCOS granulosa cell and declares the interaction between ovarian steroidogenesis and lipid metabolism and fatty acid biosynthetic process in granulosa cells, which is proved to be harmful for follicle growth and ovulation *in vitro*. Furthermore, AR signalling may be the key mediator of androgen action in hyperandrogenic environment. Overall, these results provide new insights into the mechanism of ovarian dysfunction in PCOS.

## Data Availability Statement

The data presented in the study are deposited in the GEO database, accession number GSE193123.

## Ethics Statement

The studies involving human participants were reviewed and approved by the Peking University Third Hospital Medical Science Research Ethics Committee. The patients/participants provided their written informed consent to participate in this study. The animal study was reviewed and approved by the Animal Care and Use Committee of Peking University.

## Author Contributions

BL, XQ, and CY performed experiments and analyzed data. BL and XQ prepared the figures and drafted the manuscript. JQ and YP conceived and designed the research and approved the final version of manuscript. All authors contributed to the article and approved the submitted version.

## Funding

This work was supported by the National Key Research and Development Program of China (2018YFC1003200, 2018YFC1003900), the National Natural Science Foundation of China (82022028, 81730038, 82001506), the Key Clinical Projects of Peking University Third Hospital (BYSYZD2019020), and the CAMS Innovation Fund for Medical Sciences (2019-I2M-5-001).

## Conflict of Interest

The authors declare that the research was conducted in the absence of any commercial or financial relationships that could be construed as a potential conflict of interest.

## Publisher’s Note

All claims expressed in this article are solely those of the authors and do not necessarily represent those of their affiliated organizations, or those of the publisher, the editors and the reviewers. Any product that may be evaluated in this article, or claim that may be made by its manufacturer, is not guaranteed or endorsed by the publisher.
